# Parents’ Perceptions of the Neighbourhood Built Environment Are Associated with the Social and Emotional Development of Young Children

**DOI:** 10.3390/ijerph19116476

**Published:** 2022-05-26

**Authors:** Trina Robinson, Andrea Nathan, Kevin Murray, Hayley Christian

**Affiliations:** 1 Telethon Kids Institute, The University of Western Australia, Nedlands, WA 6009, Australia; andrea.nathan@telethonkids.org.au (A.N.); hayley.christian@uwa.edu.au (H.C.); 2 School of Population and Global Health, The University of Western Australia, Nedlands, WA 6009, Australia; kevin.murray@uwa.edu.au

**Keywords:** social-emotional development, child development, neighbourhood, built environment, parent, perceptions, children

## Abstract

The influence of the neighbourhood built environment on young children’s physical development has been well-documented; however, there is limited empirical evidence of an association with social and emotional development. Parental perceptions of the neighbourhood built environment may act as facilitators or barriers to young children’s play and interactions in their local environment. The aim of this study was to examine the associations between parents’ perceptions of the neighbourhood built environment and the social-emotional development of children aged two-to-five years. Parents’ positive perceptions of traffic safety (OR 0.74; 95% CI 0.55, 0.98), crime safety (OR 0.79; 95% CI 0.64, 0.99) and land use mix–access (OR 0.74; 95% CI 0.56, 0.98) were associated with lower odds of social-emotional difficulties, while positive perceptions of walking and cycling facilities were associated with higher odds of difficulties (OR 1.26; 95% CI 1.02, 1.55). Positive perceptions of land use mix–access (OR 1.32; 95% CI 1.03, 1.69), street connectivity (OR 1.35; 95% CI 1.10, 1.66) and neighbourhood aesthetics (OR 1.27; 95% CI 1.01, 1.60) were associated with higher odds of prosocial behaviours. Interventions to improve parents’ perceptions of built environment features may facilitate opportunities for play and interactions which contribute to healthy social-emotional development.

## 1. Introduction

A child’s early years are the most important developmental phase throughout the lifespan, critical for laying the foundation for future physical and mental health and wellbeing [1]. Social-emotional development is a complex construct that forms a key component of a child’s mental health [2]. The first five years of life are a crucial time to develop social-emotional skills, such as emotional regulation, executive functioning, a readiness to explore, social competence, responsibility, and empathy. These skills determine a young child’s ability to adapt and deal with daily challenges and ultimately their capacity to lead a full and productive life [3].

Estimates of the prevalence of social-emotional difficulties in young children vary significantly depending on the study methodology, with estimates ranging from 5.5 per cent [4] to 16 per cent [5] of young children with difficulties. Importantly, social-emotional development follows a social gradient whereby increasing socioeconomic disadvantage is associated with poorer developmental outcomes, starting in children as young as two years [6], with difficulties often continuing into adulthood [7].

It is widely recognised that a child’s home is the most important environment for social-emotional development [8] as young children can spend a large amount of time in the home environment and are influenced by family relationships and parenting practices. However, the socio-ecological theory highlights that early development occurs in the context of multiple, interrelated social and physical environments [9]. The neighbourhood in which a child lives is recognised as a key influence of early child development [10,11,12]. It is important to acknowledge that a child’s exposure to their neighbourhood may be influenced by family socio-economic factors. For example, parents who work multiple jobs or full-time may have less time to spend with their child in the local neighbourhood, thus limiting children’s exposure and the influence that the neighbourhood has on their development. In addition, a recent study found that the association between neighbourhood disadvantage and behaviour problems differs by family socio-economic status whereby children from low-income households who live in more disadvantaged neighbourhoods are more likely to develop behaviour problems than children from higher-income households [13]. Thus, both family and neighbourhood socio-economic factors should be considered in child development and built environment research.

While a neighbourhood can be defined simply as the geographical area close to a person’s home, it is the components of a neighbourhood that distinguish it from other neighbourhoods, such as its cultural, political, historical, economic, social, and built characteristics. The influence of the built characteristics of a neighbourhood on child health and development has elicited substantial research over the past three decades, mostly exploring its impact on children’s physical activity levels [14,15,16]. There is, however, limited, and sometimes contradictory, evidence around the role that the neighbourhood built environment plays in early social-emotional development [16,17,18,19,20]. Built environment features, such as streets, footpaths, open public spaces, playgrounds, and child-friendly services are important as they can facilitate or hinder opportunities for play and social interaction [14,21]. An increasing body of research has investigated the role of the neighbourhood built environment on outdoor play and provides some evidence for potential pathways to social-emotional development [14]. As the neighbourhood is a major setting for children’s outdoor play, providing safe and interesting local environments may help to reduce disparities in child development [22].

Most previous research has used objective measures, such as geospatial data, to investigate associations between the neighbourhood built environment and early social-emotional development, with results indicating that the built environment has a small but significant effect on developmental outcomes [16,17,18,19]. For example, high traffic exposure [16] and greater land-use mix [17] have been associated with increased social-emotional vulnerability while the presence of an attractive local park [16] or local green space [18,23] has been associated with fewer social-emotional problems, while access to child-friendly destinations, such as child health services and schools, is associated with social competence [14].

Only assessing the objective features of a neighbourhood, however, assumes that children are exposed to, and experience, their neighbourhoods in the same way. In contrast to older children who have more autonomy to independently explore the neighbourhood, young children are dependent on their parents for such activities. It is possible that parents’ perceptions of the neighbourhood environment have a greater influence on young children’s exposure to the neighbourhood than the actual objectively measured built environment [24]. In addition to objective data about neighbourhood features, subjective data, such as parents’ perceptions of the quality and accessibility of the neighbourhood built environment, are likely to provide a more complete picture of the way children are exposed to their neighbourhood [25,26]. Previous studies investigating the relationship between objective and subjective measures of built environment features and adults’ physical activity levels have found only poor to moderate levels of agreement between objective and subjective measures of the same built environment feature [27,28,29]. This suggests that perceived and objective measures capture different constructs of the neighbourhood environment [27] and environmental interventions may not be as effective if perceptions of the environment are not also considered [30].

Most previous research investigating parent perceptions of the neighbourhood built environment focus on safety, in particular, traffic safety and fear of strangers [31,32]. The high dependence on cars to transport children has resulted in more traffic and less people on neighbourhood streets which may exacerbate parental concerns about traffic injuries and stranger abductions. While perceptions of traffic safety correlate with traffic-related injuries and deaths in children, concerns about stranger abduction are largely over-estimated and driven by fear [32]. Foster et al. [33] suggest parent fear could be alleviated by making changes to both the built (e.g., street lighting, maintenance of amenity) and social (e.g., social cohesion) neighbourhood environment.

Studies of the relationship between the perceived quality of the neighbourhood environment and early social-emotional development have produced mixed findings depending on the feature of the built environment being studied. Some studies have found no association between parents’ perceptions of the quality of the neighbourhood environment and young children’s prosocial behaviour [20,34] or externalising behaviours, such as conduct and hyperactivity [20]; however, cleaner neighbourhoods have been associated with prosocial behaviours [20]. The perceived quality of green spaces has been positively associated with all aspects of social-emotional well-being including prosocial behaviour [35], regardless of socioeconomic factors [36]. Importantly, the biggest challenge facing neighbourhood effects research is the potential for residential self-selection bias [37]. Specifically, do parents choose to live in neighbourhoods that support their attitudes and behaviour or does the neighbourhood design change their attitudes and behaviour? It is difficult to determine the direction of bias in self-selection [38] which can result in an over- or under-estimation of neighbourhood effects on child development [39].

To address these evidence gaps, we explored the relationship between parents’ perceptions of the neighbourhood built environment and early social-emotional development. Secondary research questions included: (a) Do parents’ perceptions of the neighbourhood built environment differ by level of neighbourhood socioeconomic disadvantage? and (b) Do parents’ perceptions of the neighbourhood built environment differ based on the neighbourhood they chose to live in (residential self-selection)?

## 2. Materials and Methods

### 2.1. Study Design

Cross-sectional baseline parent survey data from the Play Spaces and Environments for Children’s Physical Activity (PLAYCE) study were used. The PLAYCE baseline study (Perth metropolitan area, Western Australia; 2015 to 2018) investigated the influence of the early childhood education and care (ECEC), home and urban neighbourhood environments on physical activity, health and development of children aged two-to-five years. The study protocol has been previously published [40]. Parent surveys with missing data for socio-demographic characteristics or outcomes of interest were excluded from the analysis resulting in an analytic sample of 1492 surveys.

### 2.2. Measures

#### 2.2.1. Social-Emotional Development

Social-emotional development was reported by parents using the Strengths and Difficulties Questionnaire (SDQ) [41]. A modified version of the SDQ specifically for parents of children aged two-to-four years old [42] was used as it has been shown to have good validity for identifying psychosocial problems in young children [43]. The SDQ consists of 25 items divided into five subscales of five items each, measuring emotional problems, conduct problems, hyperactivity, peer problems, and prosocial behaviours. A total difficulties score was calculated by summing all subscale scores except the prosocial behaviour subscale, consistent with established criteria [42]. High scores on the first four subscales and the total difficulties score indicate more social-emotional difficulties while a high score for prosocial behaviour indicates more positive behaviour.

Parents rated their child’s behaviour over the past six months for each SDQ item (three-point scale: ‘not true’, ‘somewhat true’, ‘certainly true’). Mean scores were calculated for total difficulties and each subscale and then categorised into dichotomous variables (borderline/abnormal and normal) using established criteria [42].

#### 2.2.2. Perceptions of the Neighbourhood Built Environment

Parents’ perceptions of features of the neighbourhood built environment were measured using a modified version of the Neighbourhood Environment Walkability Scale for Youth (NEWS-Y) [44]. The NEWS-Y scale has acceptable test-retest reliability and construct validity, and the internal consistency of all subscales is acceptable [45].

Six of the original nine NEWS-Y subscales were used in the parent survey to measure: pedestrian and traffic safety, crime safety, land use mix–access (e.g., access to shops, parks, public transport), street connectivity, walking and cycling facilities, and neighbourhood aesthetics. Thirteen additional items were added across the subscales, based on Vanwolleghen et al.’s version [46], which specifically relate to young children, such as access to local play equipment, safety of walking with young children during the day and presence of footpaths on both sides of the street.

Each item was scored on a four-point Likert scale (strongly disagree to strongly agree) with some items reverse-coded so that all items were in the same conceptualized direction. Higher scores represented more positive perceptions of the built environment [45].

#### 2.2.3. Residential Self-Selection

To account for the potential bias created by residential self-selection, parents were asked to rate the importance of 25 reasons for choosing their current place of residence (five-point Likert scale: 1 = not at all important, 3 = somewhat important, 5 = very important). Reasons included affordability, safety, quality of local services and facilities, walkability, and proximity to schools and employment. Items were based on a similar scale developed for the International Physical Activity and Environment Network (IPEN) adolescent parent survey [47].

Due to many of the 25 residential self-selection variables measuring similar constructs, a factor analysis was conducted using principal axis factoring and oblique rotation with the goal of identifying underlying constructs. The 25 items were reduced to five residential self-selection factors which accounted for 57 per cent of the variance (Supplementary Table S1). The item ‘affordability/value’ did not load onto any factors but was retained as a single item because it was the third most important reason for parents choosing their neighbourhood. Cronbach’s alpha scores were calculated for each factor to test the reliability of the factor analysis (coefficient acceptance level set at a minimum 0.7) [48]. Two factors had low alpha scores (both 0.62) and were not included in the analysis. Mean factor scores for the three remaining factors—child friendliness, pedestrian safety from traffic, and access to shops and services—and the single item of affordability, were used as predictor variables for exploring associations between residential self-selection and parents’ perceptions of the neighbourhood built environment.

To inform the main analysis, linear regression analysis was conducted to determine if residential self-selection was a potential confounding factor in the relationship between parents’ perceptions of the neighbourhood built environment and social-emotional development (Supplementary Table S2). As significant associations were found across all subscales, residential self-selection factors were included as confounding variables in the main analysis.

#### 2.2.4. Outdoor Play

Outdoor play was reported by parents using the Outdoor Playtime Checklist [49]. Parents responded to two questions asking how much time their child spends playing outdoors on a typical day in two locations: in the yard or street around their house, and at a park, playground, or outdoor recreation area. The day was divided into three time periods: wake-up time until noon, noon until 6 p.m., and 6 p.m. until bedtime. Within each time period, the amount of outdoor play was reported using a five-point scale (0 min, 1–15 min, 16–30 min, 31–60 min, over 60 min) and were coded 0 through 4. Responses for each question were summed resulting in a score ranging from 0 to 24 [49].

#### 2.2.5. Neighbourhood Socioeconomic Disadvantage

Residential postcode was matched to administrative data from the 2016 Australian Bureau of Statistics’ (ABS) Socio-Economic Indexes for Areas (SEIFA) as a measure of postcode-level socioeconomic status for each participant [50]. The SEIFA Index of Relative Socioeconomic Disadvantage (IRSD) was used as previous research on neighbourhood effects has indicated that disadvantage affects child development outcomes, rather than socioeconomic advantage having a protective effect [51]. The IRSD is calculated using a weighted combination of variables that relate to disadvantage including low income, low educational attainment, high unemployment, long-term health condition or disability, and one-parent families [50].

Each participant postcode was assigned an IRSD score [52] and scores were then divided into deciles, as established by the ABS. Three categories were created: ‘high disadvantage’ (bottom four deciles), ‘low to moderate disadvantage’ (middle four deciles) and ‘very low disadvantage’ (top two deciles).

Neighbourhood socioeconomic disadvantage was investigated as a potential confounding factor in the relationship between parents’ perceptions of the neighbourhood built environment and social-emotional development (Supplementary Table S3). Significant associations were found; thus, neighbourhood disadvantage was included as a confounding variable in the main analysis.

#### 2.2.6. Covariates

Potential confounders included parent age [17,35], sex [17,35], education level [17,18,35,36] and employment status [17], as well as child age and sex [17,35].

### 2.3. Statistical Analyses

Multivariable binary logistic regression was undertaken with groups of independent variables sequentially added to the model to examine associations between parent perceptions of the neighbourhood built environment and early social-emotional development. Initially, Model 1 examined associations between each of the six neighbourhood perceptions subscales and the five dichotomised social-emotional development subscale responses, adjusting for parent and child socio-demographic factors. Model 2 included all variables in Model 1 and adjusted for neighbourhood socioeconomic disadvantage. Model 3 further adjusted for residential self-selection factors and Model 4 further adjusted for outdoor play.

## 3. Results

### 3.1. Sample Characteristics

Respondents were predominantly female (91%), with a mean age of 35 years (SD 5.7 years) (Table 1). Most parents worked either full-time (34%) or part-time (47%) and had a tertiary degree (57%). The average age of children was 3.3 years (SD 0.75 years) with almost half female (48%). One-fifth of parents reported their child had an abnormal or borderline SDQ total difficulties score (Table 1). This varied by SDQ sub-scale: emotional difficulties scores (18%), conduct problems (22%), hyperactivity (21%), peer problems (19%), and prosocial behaviours (30%).

Almost a quarter (24%) of young children lived in highly socioeconomically disadvantaged neighbourhoods, with 42% in neighbourhoods of low to moderate disadvantage and 34% in neighbourhoods of very low disadvantage. On average, parents scored their neighbourhood aesthetics (mean 3.19; SD 0.55), land use mix–access (mean 2.92; SD 0.51) and pedestrian and traffic safety (mean 2.90; SD 0.46) higher than other neighbourhood features, with crime safety scoring the lowest (mean 2.54; SD 0.65). Affordability was the most important reason for parents choosing their current neighbourhood (mean 3.93; SD 1.03), followed by child friendliness (mean 3.82; SD 0.77), access to shops and services (mean 3.35; SD 0.90) and pedestrian safety from traffic (mean 3.16; SD 0.91).

### 3.2. Association between Parents’ Perceptions of the Built Environment and Social-Emotional Development

**Figure 1:**
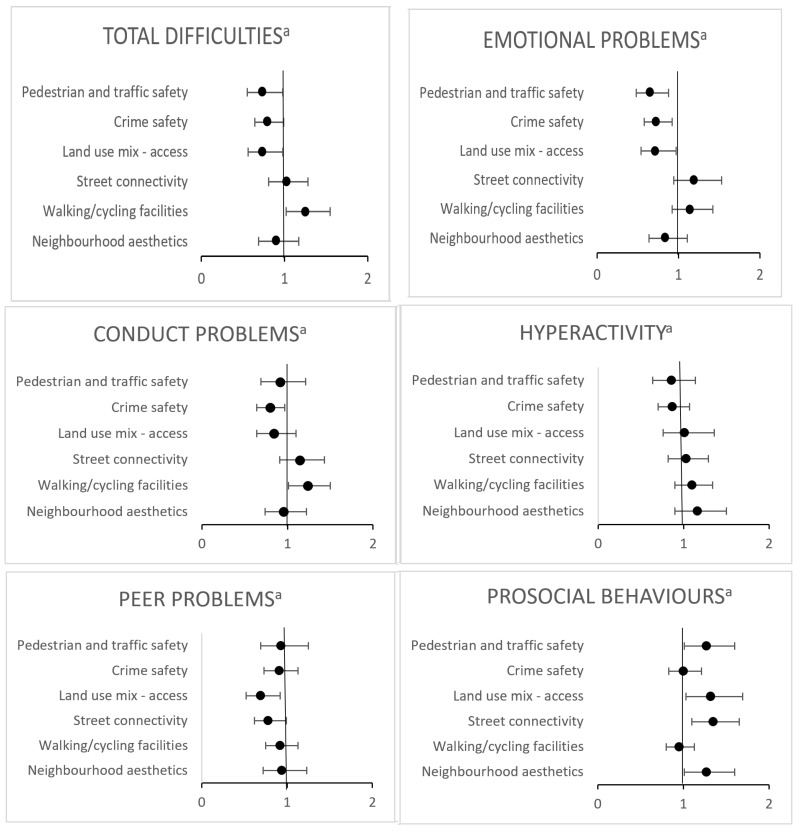
Forest plots showing odds ratios and confidence intervals of associations between parents’ perceptions of the neighbourhood built environment and social-emotional development. Statistically significant at *p* ≤ 0.05; models adjusted for parent age, sex, education, employment status and child age, sex; neighbourhood disadvantage; residential self-selection and outdoor play. ^a^ Odds ratio of having an abnormal/borderline score representing poorer development.

There was little difference in the findings with sequential adjustment for parent and child socio-demographic factors, neighbourhood socioeconomic disadvantage, residential self-selection and outdoor play; the full adjusted results (model 4) are presented in Table 2 and Figure 1.

Parents’ positive perceptions of pedestrian and traffic safety (OR 0.74; 95% CI 0.55, 0.98), crime safety (OR 0.79; 95% CI 0.64, 0.99) and land use mix–access (OR 0.74; 95% CI 0.56, 0.98) were associated with lower odds of total social-emotional difficulties. Unexpectedly, positive perceptions of walking and cycling facilities were associated with higher odds of total social-emotional difficulties (OR 1.26; 95% CI 1.02, 1.55).

Parents’ positive perceptions of pedestrian and traffic safety (OR 0.64; 95% CI 0.47, 0.87), crime safety (OR 0.73; 95% CI 0.58; 0.92) and land use mix–access (OR 0.71; 95% CI 0.53, 0.96) were associated with lower odds of emotional difficulties. Parents’ positive perceptions of crime safety (OR 0.79; 95% CI 0.64, 0.97) were associated with lower odds of conduct problems, and positive perceptions of walking/cycling facilities were associated with higher odds of conduct problems (OR 1.22; 95% CI 1.00, 1.49). Positive perceptions of land use mix–access (OR 0.69; 95% CI 0.52, 0.92) and street connectivity (OR 0.78; 95% CI 0.62, 0.99) were associated with lower odds of peer problems.

Parents’ positive perceptions of land use mix–access (OR 1.32; 95% CI 1.03, 1.69), street connectivity (OR 1.35; 95% CI 1.10, 1.66) and neighbourhood aesthetics (OR 1.27; 95% CI 1.01. 1.60) were associated with higher odds of prosocial behaviours.

## 4. Discussion

Overall, more positive parent perceptions of the neighbourhood built environment were associated with lower odds of young children’s social-emotional difficulties, independent of child and parent socio-demographic factors, neighbourhood socioeconomic disadvantage, residential self-selection and amount of outdoor play. Specifically, more positive perceptions of traffic and crime safety and access to a mix of land uses were associated with lower odds of social-emotional difficulties, and more positive perceptions of street connectivity, land use mix and neighbourhood aesthetics were associated with higher odds of prosocial behaviours.

Our finding that positive perceptions of traffic safety were related to reduced emotional difficulties, conduct problems and overall social-emotional difficulties supports previous research that found objectively measured traffic safety is related to better developmental outcomes [16,17]. Traffic may be a safety threat which can limit the amount of time children spend outside. Introducing traffic calming measures, such as roundabouts, zebra crossings and traffic lights, to residential areas is costly but has the potential to enhance road safety [53] and is associated with increased outdoor play [54]. More research is needed to determine if parents’ perceptions of traffic safety are improved as a result of introducing such measures. Parents are gatekeepers to their young children’s exposure to the local environment, and thus their perceptions of the local built environment may facilitate or be a barrier to young children’s outdoor play and interactions with adults and other children, thus providing opportunities to develop social skills and emotional resilience.

Parents’ positive perceptions of safety from neighbourhood crime were also associated with reduced emotional difficulties, conduct problems and overall social-emotional difficulties. These findings are consistent with an Australian study which found perceived levels of neighbourhood safety were associated with less conduct problems in four-to-five-year-old children [20]. Fear of child abduction when the child is playing outside in the neighbourhood is a major concern for parents, with one study finding 88% per cent of parents of five-to-six-year-olds agreed that ‘stranger danger’ was of concern to them [55]. Unlike perceptions of traffic safety which correlate with traffic-related injuries and deaths in children, parental concerns about stranger abduction are largely over-estimated and driven by fear [56]. It is therefore important to address these concerns as parental perceptions of safety are a determinant of children’s outdoor play [57]. Further research is needed to inform whether interventions should target modifications to the environment or address changing parent perceptions through, for example, raising awareness of environmental cues, such as wayfinding signage to encourage parents to explore their neighbourhood with their children. As safety concerns can be triggered by the absence of people on streets [58], initiatives which encourage more people to use the streets and public spaces may have the added benefit of providing natural surveillance of children which may in turn increase parents’ perceptions of safety in the neighbourhood.

Children with parents who perceived their neighbourhood to have better access to local services through greater land use mix had lower odds of social-emotional difficulties and higher odds of prosocial behaviours. In addition, more connected streets were associated with better prosocial behaviours and less peer problems. Previous studies have shown mixed results of the association between land-use mix and street connectivity and early child development. Our findings are consistent with Renzaho et al. [34], who also used the SDQ as an outcome measure and found that parents who perceived a lack of infrastructure and limited access to facilities reported their children had greater social-emotional difficulties. However, our findings are in contrast with Edwards and Bromfield [20], who found that perceptions of the availability of neighbourhood facilities, such as public transport, parks, shops, footpaths, and lighting, were not as important for the development of either social-emotional difficulties or prosocial behaviours, as measured by the SDQ, as other perceived neighbourhood attributes, such as safety and cleanliness. Variations across studies in how the neighbourhood was defined and the instruments used to measure neighbourhood perceptions may, in part, explain the inconsistent findings.

Our research highlights that greater street connectivity and land-use mix appear to be beneficial for early social-emotional development. Children who have more exposure to people and places through more walkable neighbourhoods are better able to develop their emotional resilience and social skills. This concurs with new urbanism planning principles which promote human-scale urban design within compact neighbourhoods with many activities of daily living within walking or cycling distance [59]. However, greater street connectivity can also increase exposure to traffic and may pose safety concerns for parents [18]. Urban planners, transport planners and local councils have a significant role to play in understanding the needs of the local community to ensure built environments are conducive to healthy child development.

More positive perceptions of neighbourhood aesthetics were associated with greater prosocial behaviours. This finding is consistent with previous research which found high-quality local green spaces [35,60] and neighbourhood cleanliness [20] were associated with young children’s prosocial behaviours as measured by the SDQ. There is substantial evidence that urban greenery has positive effects on mental health in both children and adults [61,62]. It is important to consider the perceived quality, not just access to neighbourhood green spaces, with the former more important for mental health [60,63].

Unexpectedly, positive parental perceptions of walking and cycling facilities were associated with social-emotional difficulties, which was only significant after residential self-selection was taken into account. It is unclear what may be driving this relationship; however, it highlights the importance of considering residential self-selection factors in studies examining the relationship between parent perceptions of the built environment and child development. Qualitative research could further explore and unpack the role of residential self-selection on parents’ perceptions.

The strength and significance of the models remained relatively unchanged with the addition of the child and parent socio-demographic factors, and the relationships did not attenuate after adjusting for neighbourhood disadvantage and residential self-selection. In addition, the amount of outdoor play did not explain the relationship between parents’ perceptions of the built environment and social-emotional development. This suggests that the direct relationship between perceptions of the neighbourhood built environment and social-emotional development is robust, and/or that the influence of the neighbourhood environment on social-emotional development occurs through other pathways not examined in this study (e.g., parent-child active travel which is relatively low in this young age group). Based on these findings, preliminary implications for urban planning policy and practice include the need to consider parents’ perceptions of the built environment in guidelines to create neighbourhoods that are beneficial to healthy child development. Future research should seek to determine if parents’ perceptions act as a moderator between the objective characteristics of a neighbourhood and social-emotional development and further examine these relationships in disadvantaged neighbourhoods and for those experiencing economic hardship. These findings can be used to inform whether interventions should target modifications to the environment, perceptions, or both. Further research could also examine how these findings differ by socio-demographic group and explore why some perceived neighbourhood features were significantly associated with social-emotional development outcomes but others were not.

### Strengths and Limitations

A limitation of this cross-sectional study was that it was unable to determine a temporal relationship between parent perceptions and social-emotional development. Observational research investigating associations between the neighbourhood environment and health assumes that the direction of effects flows from the neighbourhood to the individual but does not consider how individuals may influence the neighbourhood through social engagement with neighbours, upkeep of their properties or contributing to the safety of the neighbourhood. There is a need for longitudinal research to determine the nature and direction of these relationships in this young age group. Another limitation of this study includes parent-reported measures for both the predictor and outcome variables which may have introduced reporting or recall bias. In addition, the sample only included parents whose children attended an ECEC centre, with the majority of respondents being mothers (91%), which may mean the results are not fully generalisable. Fathers may perceive the neighbourhood environment and their child’s social-emotional development differently. Thus, future studies should investigate the impact of fathers’ perceptions of the neighbourhood environment on child development.

Nevertheless, study strengths included the large sample size and adjustments for child and parent socio-demographic characteristics and residential self-selection. There are temporal issues with retrospectively asking parents to state the reasons for moving into their neighbourhood in a cross-sectional study; however, our study attempted to account for over- or under-estimation of neighbourhood effects by identifying the most important reasons for selecting a neighbourhood through factor analysis and adjusting for these factors in the models.

## 5. Conclusions

This study found that parents’ perceptions of the neighbourhood built environment are associated with young children’s early social-emotional development outcomes. A better understanding of the role of parents’ perceptions of the built environment in early social-emotional development may potentially contribute to building healthier neighbourhoods for families. This is of upmost importance with a child’s early years being a critical developmental phase for future physical and mental health and wellbeing.

## Figures and Tables

**Table 1:** Sample Characteristics.

Characteristic	*n*	Mean (SD) or %
**Parent socio-demographic factors**		
Age (years)	1492	35 (5.7)
Sex (female)	1492	91%
Education		
Bachelor’s degree/Postgraduate	857	57%
Trade/Diploma	412	28%
Secondary or lower	223	15%
Employment status		
Full-time	507	34%
Part-time	697	47%
Unpaid work	28	2%
Not working/Home duties	260	17%
**Child socio-demographic factors**		
Age (years)	1492	3.3 (0.75)
Sex (female)	1492	48%
**Social-emotional development**		
Total difficulties ^a^	1490	20%
Emotional difficulties ^b^	1492	18%
Conduct problems ^b^	1492	22%
Hyperactivity ^b^	1491	21%
Peer problems ^b^	1491	19%
Prosocial behaviours ^b^	1490	30%
**Neighbourhood socioeconomic disadvantage ^c^**		
High disadvantage	352	24%
Low to moderate disadvantage	625	42%
Very low disadvantage	515	34%
**Perceptions of neighbourhood built environment ^d^**		
Pedestrian and traffic safety	1492	2.90 (0.46)
Crime safety	1492	2.54 (0.65)
Land use mix–access	1492	2.92 (0.51)
Street connectivity	1492	2.82 (0.59)
Walking and cycling facilities	1492	2.58 (0.68)
Neighbourhood aesthetics	1492	3.19 (0.55)
**Residential self-selection factors ^e^**		
Child friendliness	1492	3.82 (0.77)
Pedestrian safety from traffic	1492	3.16 (0.91)
Access to shops and services	1492	3.35 (0.90)
Affordability	1492	3.93 (1.03)
**Outdoor play ^f^**		
Total outdoor play	1426	9.16 (4.64)

^a^ Borderline/abnormal score of total difficulties (total of all subscale scores except prosocial behaviours). ^b^ Borderline/abnormal score representing poorer development. ^c^ Postal codes allocated a decile using SEIFA Index of Relative Socio-Economic Disadvantage. Deciles categorised into high, low to moderate and very low disadvantage. ^d^ Parent perception subscales 4-point Likert scale (1 = strongly disagree; 4 = strongly agree). ^e^ Mean score of residential self-selection factors; 5-point Likert scale (1 = not at all important; 5 = very important). ^f^ Total amount of time in outdoor play: Sum of 6 different time periods on a 5-point scale (0 = 0 min; 1 = 1–15 min; 2 = 16–30 min; 3 = 31–60 min; 4 ≥ 60 min) resulting in 0–24 scale.

**Table 2:** Adjusted logistic regression odds ratios examining relationships between perceived neighbourhood built environment features and social-emotional development response outcomes.

Social-Emotional Development Outcome	Perceived Neighbourhood Built Environment Feature ^a^	Model 1 ^b^ OR (95% CI) *n* = 1492	Model 2 Model 1 + n’hood Disadvantage ^d^ OR (95% CI) *n* = 1492	Model 3 Model 2 + Residential Self-Selection Factors ^e^ OR (95% CI) *n* = 1492	Model 4 Model 3 + Outdoor Play ^f^ OR (95% CI) *n* = 1426
Total social–emotional difficulties ^c^	Pedestrian/traffic safety	0.68 (0.52, 0.90) *	0.69 (0.52, 0.91) *	0.74 (0.55, 0.98) *	0.74 (0.55, 0.98) *
	Crime safety	0.76 (0.62, 0.93) *	0.76 (0.62, 0.94) *	0.76 (0.62, 0.94) *	0.79 (0.64, 0.99) *
	Land use mix–access	0.70 (0.54, 0.90) *	0.70 (0.55, 0.90) *	0.73 (0.56, 0.96) *	0.74 (0.56, 0.98) *
	Street connectivity	0.98 (0.79, 1.22)	0.98 (0.79, 1.23)	1.02 (0.81, 1.27)	1.02 (0.81, 1.28)
	Walking/cycling facilities	1.16 (0.95, 1.40),	1.17 (0.96, 1.42)	1.24 (1.02, 1.52) *	1.26 (1.02, 1.55) *
	Neighbourhood aesthetics	0.82 (0.65, 1.04)	0.83 (0.65, 1.06)	0.87 (0.67, 1.12)	0.90 (0.69, 1.17)
Emotional difficulties	Pedestrian/traffic safety	0.60 (0.45, 0.80) *	0.61 (0.46, 0.82) *	0.61 (0.45, 0.82) *	0.64 (0.47, 0.87) *
	Crime safety	0.70 (0.56, 0.86) *	0.72 (0.58, 0.89) *	0.72 (0.58, 0.90) *	0.73 (0.58, 0.92) *
	Land use mix–access	0.68 (0.52, 0.88) *	0.68 (0.53, 0.89) *	0.69 (0.52, 0.92) *	0.71 (0.53, 0.96) *
	Street connectivity	1.17 (0.93, 1.47)	1.18 (0.93, 1.48)	1.20 (0.95, 1.52)	1.22 (0.96, 1.55)
	Walking/cycling facilities	1.05 (0.86, 1.29)	1.08 (0.88, 1.32)	1.13 (0.92, 1.39)	1.15 (0.93, 1.43)
	Neighbourhood aesthetics	0.81 (0.64, 1.04)	0.84 (0.66, 1.08)	0.85 (0.65, 1.10)	0.85 (0.65, 1.12)
Conduct Problems	Pedestrian/traffic safety	0.94 (0.72, 1.23)	0.94 (0.72, 1.23)	0.97 (0.74, 1.28)	0.91 (0.69, 1.21)
	Crime safety	0.79 (0.65, 0.96) *	0.78 (0.64, 0.96) *	0.79 (0.64, 0.96) *	0.79 (0.64, 0.97) *
	Land use mix–access	0.83 (0.65, 1.06)	0.83 (0.65, 1.06)	0.86 (0.66, 1.12)	0.84 (0.64, 1.11)
	Street connectivity	1.13 (0.91, 1.40)	1.13 (0.91, 1.39)	1.16 (0.94, 1.44)	1.13 (0.91, 1.41)
	Walking/cycling facilities	1.18 (0.98, 1.41)	1.18 (0.98, 1.42)	1.23 (1.01, 1.49) *	1.22 (1.00, 1.49) *
	Neighbourhood aesthetics	0.90 (0.72, 1.14)	0.90 (0.71, 1.13)	0.94 (0.74, 1.20)	0.94 (0.73, 1.21)
Hyperactivity	Pedestrian/traffic safety	0.83 (0.62, 1.07)	0.84 (0.64, 1.10)	0.85 (0.64, 1.12)	0.86 (0.65, 1.14)
	Crime safety	0.82 (0.68, 1.01)	0.84 (0.69, 1.03)	0.85 (0.69, 1.04)	0.87 (0.70, 1.07)
	Land use mix–access	1.01 (0.78, 1.29)	1.02 (0.80, 1.31)	0.99 (0.76, 1.30)	1.00 (0.75, 1.32)
	Street connectivity	1.08 (0.87, 1.33)	1.09 (0.88, 1.35)	1.08 (0.87, 1.34)	1.05 (0.84, 1.31)
	Walking/cycling facilities	0.98 (0.96, 1.01)	1.08 (0.90, 1.31)	1.10 (0.90, 1.33)	1.11 (0.91, 1.35)
	Neighbourhood aesthetics	1.05 (0.83, 1.32)	1.09 (0.86, 1.38)	1.11 (0.87, 1.43)	1.16 (0.89, 1.50)
Peer problems	Pedestrian/traffic safety	0.82 (0.61, 1.08)	0.84 (0.63, 1.11)	0.86 (0.65, 1.15)	0.93 (0.69, 1.25)
	Crime safety	0.83 (0.68, 1.02)	0.84 (0.68, 1.04)	0.85 (0.69, 1.06)	0.91 (0.73, 1.13)
	Land use mix–access	0.64 (0.49, 0.82) *	0.64 (0.50, 0.83) *	0.65 (0.49, 0.85) *	0.69 (0.52, 0.92) *
	Street connectivity	0.79 (0.63, 0.98) *	0.79 (0.63, 0.99) *	0.81 (0.65, 1.02)	0.78 (0.62, 0.99) *
	Walking/cycling facilities	0.90 (0.74, 1.09)	0.92 (0.76, 1.12)	0.94 (0.77, 1.16)	0.92 (0.75, 1.13)
	Neighbourhood aesthetics	0.83 (0.65, 1.06)	0.87 (0.68, 1.11)	0.92 (0.71, 1.19)	0.94 (0.72, 1.23)
Prosocial behaviours	Pedestrian/traffic safety	1.37 (1.07, 1.74) *	1.37 (1.07, 1.74) *	1.25 (0.98, 1.61)	1.27 (0.99, 1.64)
	Crime safety	1.04 (0.87, 1.24)	1.04 (0.87, 1.24)	1.03 (0.86, 1.24)	1.00 (0.83, 1.21)
	Land use mix–access	1.40 (1.13, 1.74) *	1.40 (1.13, 1.75) *	1.36 (1.07, 1.73) *	1.32 (1.03, 1.69) *
	Street connectivity	1.35 (1.12, 1.64) *	1.35 (1.11, 1.64) *	1.33 (1.09, 1.62) *	1.35 (1.10, 1.66) *
	Walking/cycling facilities	1.03 (0.87, 1.21)	1.03 (0.87, 1.21)	0.94 (0.79, 1.12)	0.95 (0.80, 1.13)
	Neighbourhood aesthetics	1.36 (1.10, 1.67) *	1.37 (1.11, 1.70) *	1.29 (1.03, 1.61) *	1.27 (1.01, 1.60) *

Odds ratio (OR), Confidence Interval (CI), * Statistically significant at *p* ≤ 0.05. ^a^ 4-point Likert scale (1 = strongly disagree; 4 = strongly agree). ^b^ includes adjustments for socio-demographic variables (parent age, sex, education, employment status and child age, sex). ^c^ Odds ratio of having an abnormal/borderline score representing poorer development. ^d^ SEIFA Index of Socio-Economic Disadvantage at the postal code level; reference category is ‘very low disadvantage’. ^e^ Self-selection factors were ‘child-friendly neighbourhood’, ‘pedestrian safety from traffic’, ‘access to shops and services’, and ‘affordability’. ^f^ Total amount of time in outdoor play: Sum of 6 different time periods on a 5-point Likert scale (0 = 0 min; 4 ≥ 60 min) resulting in 0–24 scale. Model 1 includes adjustments for socio-demographic variables (parent age, child age, child sex, parent education and parent employment status). Model 2 includes adjustments for socio-demographic variables + neighbourhood socioeconomic disadvantage. Model 3 includes adjustments for socio-demographic variables + neighbourhood socioeconomic disadvantage + residential self-selection factors. Model 4 includes adjustments for socio-demographic variables + neighbourhood socioeconomic disadvantage + residential self-selection factors + outdoor play.

## Data Availability

The non-identifiable data presented in this study are available on reasonable request from the corresponding author.

## Supplementary Materials

The following supporting information can be downloaded at: https://www.mdpi.com/article/10.3390/ijerph19116476/s1, Table S1: Factor loadings of residential self-selection items; Table S2: Adjusted linear regression coefficients exploring associations between residential self-selection factors and perceived neighbourhood built environment outcomes; Table S3: Adjusted linear regression coefficients exploring associations between neighbourhood socioeconomic disadvantage and perceived neighbourhood built environment outcomes.
