# Supplementary material for: Parents’ Perceptions of the Neighbourhood Built Environment Are Associated with the Social and Emotional Development of Young Children
Source: Int J Environ Res Public Health. 2022 May 26;19(11):6476. doi: 10.3390/ijerph19116476 (PMC9180167; doi:10.3390/ijerph19116476)
Supplement: Supplementary file 1 [file ijerph-19-06476-s001.zip › ijerph-1681917-supplementary.pdf]

Table S1. Factor loadings of residential self-selection items (*n*=1492)

**Item**	**Factor 1** Child friendly	**Factor 2** Traffic safety	**Factor 3** Access to shops and services	**Factor 4** Land use mix	**Factor 5** Proximity to leisure facilities
Quality of schools	0.98				
Safety from crime	0.70				
Closeness to school	0.68				
Neighbourhood designed to be safe for children	0.63				
Sense of community	0.57				
Presence of other children in neighbourhood		0.46			
Streets designed to be safe for pedestrians/cyclists		0.90			
Streets have footpaths		0.87			
Streets designed to minimise traffic		0.81			
Distance from busy street		0.49			
Closeness to shops/services			0.90		
Closeness to public transport			0.84		
Ease of walking			0.58		
Access to freeway				0.66	
Closeness to healthcare facilities				0.56	
Closeness to beach					0.76
Closeness to variety of parks					0.74
Mean factor score (SD)	3.82(0.77)	3.16(0.90)	3.35(0.90)	3.02(0.98)	3.11(1.01)
Cumulative % of variance	33.75	41.08	52.19	52.19	57.04
Cronbach's α	0.84	0.85	0.62	0.62	0.62

Table S2. Adjusted linear regression coefficients exploring associations between residential self-selection factors and perceived neighbourhood built environment outcomes (*n*=1492)

**Residential self-selection factor**	**Pedestrian & traffic safety**[a] *B* (95% CI)	**Crime safety**[a] *B* (95% CI)	**Land use mix – access**[a] *B* (95% CI)	**Street connectivity**[a] *B* (95% CI)	**Walking/cycling facilities**[a] *B* (95% CI)	**N'hood aesthetics**[a] *B* (95% CI)
Child friendliness	0.12 (0.10, 0.16)*	0.06 (0.02, 0.11)*	0.10 (0.07, 0.13)*	0.07 (0.03, 0.11)*	0.17 (0.12, 0.21)*	0.21 (0.18, 0.24)*
Pedestrian safety from traffic	0.10 (0.07, 0.12)*	0.01 (-0.03, 0.04)	0.05 (0.03, 0.08)*	0.03 (0.00, 0.06)*	0.16 (0.12, 0.20)*	0.09 (0.06, 0.12)*
Access to shops and services	0.07 (0.04, 0.09)*	0.01 (-0.02, 0.05)	0.21 (0.18, 0.24)*	0.11 (0.07, 0.14)*	0.18 (0.15, 0.22)*	0.12 (0.09, 0.15)*
Affordability	-0.10 (-0.03, 0.01)	-0.08 (-0.11,-0.05)*	0.00 (-0.02, 0.03)	0.01 (-0.02, 0.04)	-0.01 (-0.05,0.02)	-0.05 (-0.08,-0.02)*

B coefficient represents the estimated change in mean associated with a one unit increase in the residential self-selection factor
* Statistically significant at $p \leq 0.05$; models adjusted for parent age, sex, education, employment status and child age, sex
[a] 4-point Likert scale (1=strongly disagree; 4=strongly agree)

Table S3. Adjusted linear regression coefficients exploring associations between neighbourhood socioeconomic disadvantage and perceived neighbourhood built environment outcomes (*n*=1492)

**Neighbourhood socioeconomic disadvantage**	**Pedestrian & traffic safety**[a] *B* (95% CI)	**Crime safety**[a] *B* (95% CI)	**Land use mix – access**[a] *B* (95% CI)	**Street connectivity**[a] *B* (95% CI)	**Walking/cycling facilities**[a] *B* (95% CI)	**N'hood aesthetics**[a] *B* (95% CI)
Very low disadvantage[b]	reference	reference	reference	reference	reference	reference
Low-moderate disadvantage[b]	-0.06 (-0.12,-0.01)*	-0.19 (-0.26,-0.11)*	-0.13 (-0.19,-0.07)*	0.10 (0.03, 0.17)*	-0.01 (-0.09, 0.08)	-0.14 (-0.21,-0.08)*
High disadvantage[b]	-0.11 (-0.18,-0.05)*	-0.34 (-0.43, -0.26)*	-0.10 (-0.17,-0.03)*	0.06 (-0.14, 0.02)	-0.24 (-0.32, -0.16)*	-0.30 (-0.37,-0.23)*

B coefficient represents the estimated difference in mean to the reference category.
* Statistically significant at $p \leq 0.05$; models adjusted for parent age, sex, education, employment status and child age, sex
[a] 4-point Likert scale (1=strongly disagree; 4=strongly agree)
[b] SEIFA Index of Socio-Economic Disadvantage at the postal code level; reference category is 'very low disadvantage'
